# Factor structure and measurement invariance of a neuropsychological test battery designed for assessment of cognitive functioning in older Mexican Americans

**DOI:** 10.1016/j.dadm.2018.08.003

**Published:** 2018-08-30

**Authors:** Brandon E. Gavett, Katie Stypulkowski, Leigh Johnson, James Hall, Sid E. O'Bryant

**Affiliations:** a Department of Psychology, University of Colorado Colorado Springs, Colorado Springs, CO, USA; b Center for Alzheimer's & Neurodegenerative Disease Research, Institute for Healthy Aging, University of North Texas Health Sciences Center, Fort Worth, TX, USA

**Keywords:** Cross-cultural comparison, Bias, Aging, Cognition

## Abstract

**Introduction:**

The present study sought to investigate the measurement invariance of commonly used neuropsychological tests in an ethnically (Hispanic vs. non-Hispanic) and linguistically (Spanish vs. English) diverse sample.

**Methods:**

Participants were 736 middle-aged and older adults (*M*_Age_ = 62.1, SD = 9.1) assessed at baseline. Measurement invariance testing was performed using multiple-group confirmatory factor analysis.

**Results:**

A five-factor model (memory, attention/executive functioning/processing speed, language, visuospatial, and motor) fit the data well (CFI = 0.979, RMSEA = 0.047) and the composite reliability of the factors ranged from .76 (visuospatial) to .97 (motor). The five-factor model was found to possess strict measurement invariance for ethnicity and language without a decrement in fit compared to a strong (scalar) invariance model (ΔCFI = .000, ΔRMSEA = .002).

**Discussion:**

These results indicate that a five-factor model is suitable for estimating cognitive functioning in Mexican Americans and non-Hispanic whites without bias by ethnicity or language.

## 1 Background

Assessment of cognitive functioning is an essential tool in cognitive aging and neurodegenerative disease research. For instance, cognitive test scores often serve as outcome variables when studying group differences, rates of change over time, or when evaluating the impact of an intervention [1], [2]. Furthermore, neuropsychological tests are often used to make inferences about the absence or presence of a latent pathological process, such as Alzheimer's disease or to assist with differential diagnosis among competing possibilities [3]. Clinical neuropsychologists rely heavily on cognitive test scores to identify a patient's strengths and weaknesses and use these results to make targeted recommendations for intervention and care [4]. Neuropsychological assessment is a noninvasive method for capturing useful information about the behavioral manifestations of an underlying neurodegenerative disease.

Although neuropsychological assessment is useful for understanding patterns of cognitive decline, this pursuit can be complicated by the considerable heterogeneity in cognitive phenotypes, not only across different neurodegenerative conditions, but across groups of individuals who differ along one or more dimensions [5], [6]. In other words, factors such as racial and ethnic diversity are associated with differences in the clinical presentations of those with both normal and pathological aging. When examining group differences in cognitive test results, it is important to distinguish between true differences in ability and differences that arise due to measurement error. Bias refers to error that varies systematically with other grouping variables. However, group differences in cognitive test results are not in and of themselves reflective of bias; differences in life history variables like education and environmental enrichment can cause real differences in cognitive abilities that may be validly captured by test score differences [7], [8], [9], [10]. Therefore, to help disentangle true differences in ability from systematic error variance, it is essential to validate the ability of a cognitive battery to make unbiased measurements of cognition across diverse groups.

Measurement invariance is the term used to describe the ability of a test score to estimate an underlying trait with equal validity across groups or over time [11], [12]. The specific types of measurement invariance and their mathematical properties have been described in detail elsewhere [12], [13], [14]. Because of the importance of making comparisons of group mean differences, we sought to determine whether a comprehensive battery of cognitive tests possesses at least “strong” (scalar) invariance for estimating cognitive functioning across different groups. This type of invariance testing uses confirmatory factor analysis (CFA) to constrain the model's factor loadings and intercepts to be equal across groups. If such a model provides a good fit to the data that are not substantially worse than a less constrained model (“weak,” or metric, invariance, which only applies equality constraints to factor loadings), then differences in group means on the factors being estimated can be interpreted validly [15].

The goal of the present study is to examine the measurement invariance properties of a cognitive test battery used in the assessment of Mexican Americans and non-Hispanic white Americans when administered in either Spanish or English. We seek to determine the type of invariance that can be achieved with this battery. Strict or strong invariance would allow for the battery to be used to compare differences in group means, whereas weak or configural invariance would suggest the possibility that group mean comparisons could be systematically affected by bias, thus not allowing valid group mean comparisons to be made. Our approach to establishing the factor structure and measurement invariance of this battery was modeled after the work of Park et al. [16] with the Alzheimer's Disease Neuroimaging Initiative neuropsychological battery.

## 2 Methods

### 2.1 Participants

Participants were 741 volunteers, aged 50 years and older, in the Health and Aging Brain among Latino Elders (HABLE) study who provided informed consent to participate in this research. More specific details about the HABLE study have been published previously [17], [18], [19], [20]. Briefly, the HABLE study is a community-based epidemiological study that focuses on understanding cognitive changes in a predominantly Mexican American sample recruited from Tarrant county, Texas. As part of this larger ongoing study, participants undergo a review of medical history, medications, and health behaviors; neuropsychological assessment; blood collection; and a medical evaluation that includes a review of systems, Hachinski Ischemic Score, and neurological examination. Participants were neither excluded from the parent HABLE study nor the present study on the basis of dementia severity. The evaluation was completed in English or Spanish depending on the participant's preference, using standard versions of each test, described in Section 2.3 below.

### 2.2 Procedure

From the initial sample of 741 participants, we identified three predominant ethnicity/language subgroups: those with Hispanic ethnicity whose primary language was English (*n* = 110), those with Hispanic ethnicity whose primary language was Spanish (*n* = 489), and those with non-Hispanic ethnicity whose primary language was English (*n* = 137). There was only one participant who reported non-Hispanic ethnicity and Spanish as primary language; because there were no other individuals with this ethnicity/language combination in the sample, this participant was excluded. An additional four participants were excluded because they reported English as their first language but were tested in Spanish, resulting in a total sample size of 736 for analysis.

### 2.3 Measures

Neuropsychological assessment was performed using the following instruments, collected at participants' baseline study visit.

#### 2.3.1 Consortium to Establish a Registry for Alzheimer's Disease List Learning

The Consortium to Establish a Registry for Alzheimer's Disease (CERAD) list learning task provides measures of immediate and delayed verbal memory. In this test, participants are shown a list of 10 words at the rate of one word every 2 seconds. Immediately after, they are asked to recall as many words as possible. This is completed three times, and the order of word presentation changes with each trial. The sum across the three learning trials is the variable used for immediate memory in the present study (CERAD total). Following a brief delay, participants are again asked to recall as many words as they can remember from the list (CERAD delayed recall). Finally, participants are asked to complete a recognition task where familiarity with target items and foils is endorsed or denied (CERAD recognition) [21].

#### 2.3.2 Wechsler Memory Scale-III Logical Memory I and II

This test provides a measure of verbal auditory memory and delayed retention. Participants are presented with short stories read aloud and are instructed to try to remember the stories exactly as they are told. They are then asked to repeat the stories back as best as they can remember (Logical Memory I). After a delay of approximately 30 minutes, participants are again asked to recall as much of the stories as they can (Logical Memory II) [22].

#### 2.3.3 Wechsler Adult Intelligence Scale-III Digit Span

This test contains two components: forward and backward Digit Span. In the forward portion of the test, participants are read a series of numbers and asked to repeat them back exactly as heard. The backward portion presents participants with a series of numbers and asks them to repeat the numbers back in reverse order. This subtest provides measures of working memory and attention [23].

#### 2.3.4 Executive Interview

This is a screening measure of executive functioning abilities and neurological soft signs that can be used to identify individuals with executive functioning deficits that may be associated with increased risk of functional deficits. The executive interview is a clinician-administered scale with 25 items, with each item scored as 0 (intact), 1 (mild deficits), or 2 (severe deficits). Higher scores reflect more pronounced deficits in executive functioning abilities. Scores were recoded to match the direction of other data (higher scores reflecting better performance) by subtracting each participant's observed score from the maximum observed score in the sample (27) [24].

#### 2.3.5 Trail Making Test

Trail Making Test parts A and B provide information on processing speed, mental flexibility, and executive functioning. Part A provides the participant with a sheet of paper displaying circles containing the numbers 1–25 in an array spread over the page. The participant is asked to connect the numbered circles in order as quickly as possible, beginning at 1 and ending at 25. Part B is similar, but includes letters and numbers. Participants are asked to draw the lines while alternating between numbers and letters. For both tests, completion time is the primary outcome measure; scores were recoded to match the direction of other data (higher scores reflecting better performance) by subtracting each participant's observed score from the maximum observed score (217″ for A and 377″ for B) [25].

#### 2.3.6 Boston Naming Test

The Boston Naming Test is a test of confrontation naming where up to 60 line-drawn pictures of common objects are presented to examinees who are asked to provide the name for each picture. Higher scores represent better naming ability [26].

#### 2.3.7 FAS and Animal Fluency

The FAS test provides the participant with a letter (F) and then asks them to name as many different words beginning with that letter as they can in 1 minute. This is then repeated with the letters A and S. The total number of words generated across the three trials–excluding proper nouns and the same words with different endings–is the outcome variable of interest. Similarly, the Animal Fluency test asks the participant to name as many animals as they can within 1 minute.

#### 2.3.8 CLOX: An Executive Clock Drawing Task

This test contains two parts. In CLOX1, the participant is presented with a blank piece of paper and is instructed to draw a clock with the hands set to 1:45. In CLOX2, the participant watches the examiner draw a clock that is set to 1:45 inside a circle. The participant is then asked to copy the clock. Higher scores represent better performance [27].

#### 2.3.9 Grip Strength Test

A hand dynamometer is used to measure grip strength in participants' hands bilaterally. Two trials per hand are administered to obtain a measure of gross motor function. Higher values represent greater grip strength [25].

### 2.4 Model selection

To identify the most appropriate model to be subjected to measurement invariance testing, we hypothesized a model based on the specific test scores available in our battery and the published literature pertaining to the cognitive domains underlying neuropsychological test performance [28], [29] with particular emphasis on a similar study performed using the Alzheimer's Disease Neuroimaging Initiative neuropsychological data [16]. The hypothesized model contained five factors: (1) memory, (2) attention/executive functioning/processing speed, (3) language, (4) visuospatial, and (5) motor. We modeled residual correlations between indicator variables sharing method variance for the three CERAD variables with one another, the two Logical Memory variables with each other, FAS with animal fluency, the two Trail Making Test variables with each other, the two Digit Span variables with one another, and the same-handed Grip Strength variables with one another. To judge the quality of model fit, we relied on the comparative fit index (CFI), Tucker-Lewis index (TLI), root mean square error of approximation (RMSEA), and standardized root mean square residual (SRMR). Good fit is indicated by CFI and TLI values ≥.95, RMSEA values (including 90% confidence intervals) < .06, and SRMR values < .09 [30]. All models described in this study were run using a robust full information maximum likelihood estimator and all indicator variables were standardized before being analyzed.

### 2.5 Measurement invariance testing

After ensuring good model fit in the entire sample, we examined model fit in each of the three groups separately to ensure a reasonably good fitting model in each group before proceeding with more formal measurement invariance testing. Our approach to measurement invariance testing followed typical procedures, in that we began by specifying a configural invariance model and then incrementally applying increasingly restrictive equality constraints across groups. The procedure involved moving from configural to metric (weak) invariance, scalar (strong) invariance, and finally strict (residual variance) invariance models. Meaningful changes in model fit were judged by ΔCFI values of .01 [13], [31] or greater and the likelihood ratio *χ*^2^ difference test [32]. Data were analyzed using Mplus version 8 [33] and R version 3.4.2 [34], including the lavaan (version 0.5–23.1097) [35] and semTools (version 0.4−14) [36] packages.

## 3 Results

Participant demographic data, dementia outcome variables, and neuropsychological test scores are shown in Table 1. In the total sample, ages ranged from 50 to 100 years and years of education ranged from 0 to 20 years. The three ethnic/language groups differed in age, education, gender composition, and scores on the Mini-Mental State Examination. All neuropsychological test scores differed across groups as well. The groups did not differ in dementia severity, as measured by the Clinical Dementia Rating.

**Table 1:** Participant demographics, dementia severity outcomes, and cognitive test scores

Variable	NH/E	H/E	H/S	Total	Effect size^a^ [95% CI]		
					NH/E versus H/E	NH/E versus H/S	H/E versus H/S
N	137	110	489	736	--	--	--
Age; M (SD)	64.47 (10.64)	61.45 (8.14)	61.53 (8.68)	62.07 (9.06)	0.31∗ [0.06, 0.57]	0.32∗ [0.13, 0.51]	−0.01 [−0.22, 0.20]
Education; M (SD)	14.60 (3.16)	11.58 (3.41)	7.20 (4.37)	9.23 (5.02)	0.92∗ [0.66, 1.19]	1.79∗ [1.57, 2.00]	1.04∗ [0.83, 1.26]
Male gender; N (%)	50 (36.5)	33 (30.0)	118 (24.1)	201 (27.3)	0.75 [0.42, 1.32]	0.55∗ [0.36, 0.85]	0.74 [0.46, 1.21]
CDR Global; M (SD)	0.20 (0.48)	0.10 (0.34)	0.20 (0.43)	0.18 (0.43)	0.23 [−0.03, 0.49]	0.01 [−0.18, 0.20]	−0.22∗ [−0.43, −0.01]
CDR Sum of Boxes; M (SD)	0.88 (2.65)	0.34 (1.75)	0.79 (2.25)	0.74 (2.27)	0.23 [−0.03, 0.49]	0.04 [−0.16, 0.23]	−0.21 [−0.42, 0.00]
MMSE; M (SD)	27.32 (3.70)	26.83 (3.73)	24.91 (4.24)	25.65 (4.2)	0.13 [−0.12, 0.39]	0.58∗ [0.39, 0.78]	0.46∗ [0.25, 0.67]
CERAD Total; M (SD)	19.99 (5.35)	18.86 (3.88)	16.16 (4.59)	17.29 (4.91)	0.24 [−0.02, 0.49]	0.80∗ [0.60, 1.00]	0.60∗ [0.39, 0.82]
CERAD DR; M (SD)	6.26 (2.61)	6.09 (2.06)	4.80 (2.44)	5.27 (2.51)	0.07 [−0.19, 0.33]	0.59∗ [0.04, 0.79]	0.55∗ [0.33, 0.76]
CERAD Recognition; M (SD)	19.23 (1.84)	19.54 (0.89)	18.35 (2.19)	18.70 (2.04)	−0.21 [−0.46, 0.05]	0.42∗ [0.22, 0.61]	0.59∗ [0.38, 0.8]
Logical Memory I; M (SD)	34.18 (13.44)	33.13 (10.93)	29.76 (11.88)	31.07 (12.18)	0.08 [−0.17, 0.34]	0.36∗ [0.17, 0.55]	0.29∗ [0.08, 0.50]
Logical Memory II; M (SD)	20.29 (10.29)	19.92 (8.63)	17.45 (8.92)	18.34 (9.22)	0.04 [−0.22, 0.29]	0.31∗ [0.12, 0.50]	0.28∗ [0.07, 0.49]
DS Forward; M (SD)	9.34 (2.32)	7.64 (1.88)	6.18 (1.51)	6.98 (2.13)	0.80∗ [0.53, 1.06]	1.84∗ [1.62, 2.06]	0.92∗ [0.71, 1.14]
DS Backward; M (SD)	5.83 (2.38)	4.63 (1.98)	3.91 (1.77)	4.37 (2.06)	0.54∗ [0.28, 0.80]	1.00∗ [0.8, 1.20]	0.40∗ [0.19, 0.61]
EXIT25; M (SD)	7.45 (5.82)	8.51 (4.12)	10.23 (4.94)	9.74 (4.96)	−0.23 [−0.67, 0.22]	−0.55∗ [−0.94, −0.17]	−0.36∗ [−0.63, −0.09]
TMT-A Time; M (SD)	47.82 (26.05)	43.01 (17.66)	70.28 (36.19)	61.98 (34.31)	0.21 [−0.05, 0.47]	−0.66∗ [−0.85, −0.46]	−0.81∗ [−1.03, −0.60]
TMT-B Time; M (SD)	124.81 (77.57)	123.51 (65.62)	168.97 (78.46)	151.91 (79.19)	0.02 [−0.25, 0.29]	−0.56∗ [−0.77, −0.36]	−0.60∗ [−0.83, −0.37]
Boston Naming Test; M (SD)	51.17 (12.07)	46.57 (9.27)	38.89 (9.93)	40.96 (10.69)	0.46∗ [0.02, 0.89]	1.22∗ [0.83, 1.6]	0.78∗ [0.53, 1.03]
FAS Fluency; M (SD)	34.19 (13.05)	27.20 (10.46)	23.40 (10.38)	25.96 (11.68)	0.58∗ [0.32, 0.84]	0.98∗ [0.78, 1.18]	0.37∗ [0.16, 0.57]
Animal Fluency; M (SD)	17.04 (5.69)	16.58 (4.50)	14.99 (4.78)	15.61 (4.99)	0.09 [−0.17, 0.34]	0.41∗ [0.22, 0.60]	0.34∗ [0.13, 0.55]
CLOX1; M (SD)	11.27 (2.56)	11.37 (2.30)	10.40 (2.60)	10.7 (2.59)	−0.04 [−0.3, 0.21]	0.34∗ [0.15, 0.53]	0.38∗ [0.17, 0.59]
CLOX2; M (SD)	13.31 (1.68)	13.44 (1.31)	12.91 (1.88)	13.06 (1.78)	−0.08 [−0.34, 0.17]	0.22∗ [0.03, 0.41]	0.29∗ [0.09, 0.50]
Grip Strength D1; M (SD)	63.93 (23.17)	55.92 (19.39)	56.88 (17.68)	58.07 (19.27)	0.37∗ [0.11, 0.63]	0.37∗ [0.18, 0.56]	−0.05 [−0.26, 0.16]
Grip Strength D2; M (SD)	66.52 (22.90)	56.65 (20.79)	58.89 (17.98)	60.00 (19.66)	0.45∗ [0.19, 0.71]	0.4∗ [0.21, 0.59]	−0.12 [−0.33, 0.09]
Grip Strength ND1; M (SD)	61.37 (22.30)	52.94 (16.69)	53.57 (16.48)	54.96 (18.00)	0.42∗ [0.16, 0.68]	0.43∗ [0.24, 0.63]	−0.04 [−0.25, 0.17]
Grip Strength ND2; M (SD)	61.27 (21.59)	53.53 (17.29)	54.43 (16.84)	55.59 (18.09)	0.39∗ [0.13, 0.65]	0.38∗ [0.19, 0.57]	−0.05 [−0.26, 0.16]

Abbreviations: CI, confidence interval; NH/E, Non-Hispanic English; H/E, Hispanic English; H/S, Hispanic Spanish; CDR, Clinical Dementia Rating; MMSE, Mini-Mental State Examination; DS, digit span; CLOX, Executive Clock Drawing Test; TMT, Trail Making Test; CERAD, consortium to establish a registry for Alzheimer's disease; DR, delayed recall; EXIT25, executive interview; D, dominant hand; ND, nondominant hand.

∗ *P* < .05.

a Effect size for male gender is the odds ratio resulting from Fisher's Exact Test. All other effect sizes are reported as Cohen's *d*.

The five-factor model fit the data well in the entire sample, CFI = .979, TLI = .973, RMSEA = 0.047 (95% confidence interval [0.041, 0.053]), SRMR = 0.028. The factor loadings derived from this model are shown in Table 2 and the estimated factor correlations are shown in Table 3. In addition to the good overall model fit, all factor loadings were strong (>.40) and in the expected direction. All factors were strongly positively correlated, with the exception of the Motor factor, which had a weak positive correlation with the other four factors. The model also fit reasonably well in each of the three ethnicity/language subgroups, as shown in Table 4, confirming the appropriateness of further measurement invariance testing.

**Table 2:** Standardized parameter estimates for five-factor model

Factor	Indicator	Estimate	SE	Est/SE	*P*
Memory	CERAD total	0.756	0.024	30.88	<.001
	CERAD recall	0.672	0.028	24.19	<.001
	CERAD recognition	0.593	0.052	11.42	<.001
	Logical Memory I	0.766	0.021	36.03	<.001
	Logical Memory II	0.747	0.023	33.14	<.001
AEPS	Digit Span F	0.543	0.026	20.85	<.001
	Digit Span B	0.643	0.022	29.70	<.001
	EXIT25	0.781	0.024	32.49	<.001
	TMT-A	0.799	0.021	38.06	<.001
	TMT-B	0.866	0.016	53.82	<.001
Language	BNT	0.865	0.020	44.16	<.001
	FAS	0.709	0.024	29.63	<.001
	Animals	0.730	0.024	30.54	<.001
Visuospatial	CLOX1	0.756	0.029	26.31	<.001
	CLOX2	0.815	0.025	33.05	<.001
Motor	Grip Strength—D1	0.932	0.028	33.06	<.001
	Grip Strength—D2	0.951	0.028	34.07	<.001
	Grip Strength—ND1	0.942	0.027	34.78	<.001
	Grip Strength—ND2	0.943	0.026	35.62	<.001

Abbreviations: AEPS, attention, executive functioning, and processing speed; BNT, Boston Naming Test; SE, standard error; CERAD, Consortium to Establish a Registry for Alzheimer's Disease; F, forward; B, backward; EXIT25, Executive Interview; TMT-A, Trail Making Test part A; TMT-B, Trail Making Test part B; FAS, letter fluency for letters F, A, and S; CLOX, Executive Clock Drawing Test parts 1 and 2; D, dominant; ND, nondominant.

**Table 3:** Factor correlation matrix in full sample

Factor	Memory	AEPS	Language	Visuospatial	Motor
Memory	1.000				
AEPS	.896	1.000			
Language	.891	.908	1.000		
Visuospatial	.753	.869	.741	1.000	
Motor	.198	.308	.327	.252	1.000

Abbreviation: AEPS, attention, executive functioning, and processing speed.

**Table 4:** Model fit in each group separately

Group	N	*χ*^*2*^	*df*	*P*	CFI	TLI	RMSEA [90% CI]	SRMR
Hispanic/English	110	152.6	133	0.12	0.985	0.981	0.037 [0.000, 0.061]	0.053
Hispanic/Spanish	488	253.7	133	<.01	0.982	0.977	0.043 [0.035, 0.051]	0.029
Non-Hispanic/English	136	218.9	133	<.01	0.963	0.952	0.069 [0.052, 0.085]	0.071

Abbreviations: CFI, comparative fit index; TLI, Tucker-Lewis Index; RMSEA, root mean square error of approximation; CI, confidence interval; SRMR, standardized root mean square residual.

As can be seen in Table 5, all of the constrained models fit the data well and there was no decrement in model fit when additional equality constraints were applied. Therefore, the results indicate that the five-factor model can be considered to have strict measurement invariance across the ethnic and language groups used in this study.

**Table 5:** Measurement invariance test results based on ethnicity/language

Model	*df*	AIC	BIC	*χ*^*2*^	CFI	RMSEA	Δ*χ*^*2*^	Δ*df*	*P*	ΔCFI	ΔRMSEA
Configural	399	26,499	27,548	615.77	0.978	0.049	--	--	--	--	--
Weak	437	26,457	27,330	649.06	0.981	0.044	25.08	38	.947	0.002	0.005
Strong	465	26,408	27,153	656.63	0.983	0.040	7.52	28	1.000	0.002	0.004
Strict	503	26,394	26,964	718.54	0.983	0.039	42.11	38	.298	0.000	0.002
Strict + Means	513	26,377	26,901	721.57	0.983	0.038	3.09	10	.979	0.001	0.001

Abbreviations: AIC, Akaike Information Criterion; BIC, Bayesian Information Criterion; CFI, comparative fit index; RMSEA, root mean square error of approximation.

The most constrained measurement invariance model (“strict + means”) applied group equality constraints to the factor means and found no decrement in fit compared to a less-constrained (strict) model. As such, three groups are considered to have equal global factor means for all five factors when these factors are estimated using a latent variable framework. In other words, when the factors are estimated without measurement error, such as with CFA, there is no evidence of group mean differences on those factors.

Finally, the composite reliability of the single-factor model was calculated using Raykov's approach [37]. The composite reliabilities (true score variance divided by observed score variance) of the five factors in the entire sample and in the subsamples are shown in Table 6. The visuospatial factor was consistently the least reliable, with composite reliabilities below the recommended threshold of *r* = .80 [38] but above .70. In contrast, the motor factor was consistently the most reliable (>.96). For the most part, the remaining factors possessed high reliability, especially in the total sample, but there were a few cases of lower than desirable reliability for some of the factors in the subsamples (see Table 6).

**Table 6:** Composite reliabilities [95% confidence intervals] for the five factors in the subsamples and the total sample

Factor	NH/E	H/E	H/S	Total
Memory	.882 [.837, .926]	.703 [.571, .835]	.821 [.780, .861]	.834 [.802, .865]
AEPS	.880 [.831, .929]	.801 [.715, .887]	.844 [.817, .871]	.860 [.841, .878]
Language	.808 [.704, .912]	.752 [.659, .846]	.797 [.761, .833]	.815 [.787, .843]
Visuospatial	.717 [.534, .899]	.718 [.526, .910]	.777 [.724, .829]	.764 [.715, .813]
Motor	.984 [.964, 1.00]	.962 [.943, .981]	.969 [.963, .975]	.969 [.965, .974]

Abbreviations: NH/E, Non-Hispanic English; H/E, Hispanic English; H/S, Hispanic Spanish; AEPS, attention, executive functioning, and processing speed.

## 4 Discussion

The current results indicate that a five-factor model of cognition—including the factors of memory; attention, executive functioning, and processing speed; language; visuospatial; and motor, as measured by 19 different neuropsychological variables–is capable of measuring cognitive functioning with equal validity in this diverse sample of participants regardless of ethnicity (Hispanic/non-Hispanic) or primary language (Spanish/English). Because this model demonstrated strict invariance, group comparisons can be made with confidence that the CFA model is capable of providing a valid estimate of actual differences in global cognitive functioning and are not influenced by systematic bias to a meaningful degree. The current results provide a strong first step toward establishing the measurement invariance of a comprehensive model for cognitive functioning in older adults from both Hispanic and non-Hispanic ethnic groups and who speak either Spanish or English. Importantly, our results suggest that these five cognitive factors can essentially be measured without bias using commonly used neuropsychological tests that were developed in English but were administered to many in Spanish. These findings can be further bolstered by replication in independent samples and in samples that contain additional racial, ethnic, and linguistic heterogeneity.

Measuring cognitive functioning with equal validity across diverse groups is an essential requirement of neuropsychological assessment instruments used in cross-cultural and cross-linguistic research and clinical practice. Considering the growing diversity of the U.S. population, more research is needed to determine whether commonly used neuropsychological assessment instruments are capable of measuring cognitive functioning with equal validity in groups that differ on important dimensions such as ethnicity and language. This is especially important for work in the area of cognitive aging, where clinicians and researchers are increasingly likely to assess patients and research participants from diverse backgrounds, where the potential introduction of systematic measurement error could affect the construct validity of the instruments used to measure cognition. Research has indicated that life history variables that covary on dimensions of race and ethnicity are important contributors to cognitive functioning in older adults [7], [8], [9], [10]. In addition, dementia onset is earlier in Mexican Americans compared to non-Hispanic whites [39], [40], [41], and the brain variables driving cognitive decline can differ based on race and ethnicity [42]. In particular, racial and ethnic differences in rates of diabetes and depression are likely to contribute to health disparities in minority groups [19], [43], [44], [45], [46]. Such findings highlight the importance of ensuring that observed differences on cognitive tests are valid and not simply a reflection of test bias.

In this study, 10 different tests—together yielding a total of 19 outcome variables—were used to derive estimates of cognitive functioning in five neuropsychological domains. Not only were these factors invariant to the ethnic and linguistic group differences in our sample, the factors were also found to possess, for the most part, high reliability. Although the focus of the current article was on measurement invariance, this finding of high composite reliability is important as well, as it indicates that the current model is capable of estimating cognitive functioning with high precision, which is another important attribute for examining group differences [47]. Future research can make use of this model to provide an essentially unbiased estimate of cognition across five domains for the purpose of studying cognitive aging in older Spanish and English-speaking Mexican Americans and English-speaking Caucasian Americans.

Although the observed neuropsychological test scores often differed across ethnic and language groups (Table 1), our results show that the five underlying cognitive factors did not significantly differ across groups. This finding is likely due to the fact that CFA was used to derive estimated factor means, which—in the context of a latent variable model—are not affected by measurement error. In contrast, the observed test scores shown in Table 1 reflect a combination of trait ability plus measurement error. Therefore, when using these tests to estimate the five factors in our model, it is necessary to use CFA to avoid the potentially biasing effects of measurement error. Failing that, application of demographically corrected normative data to the observed scores may help attenuate the effects of systematic bias on the observed test results to some extent [48], [49].

Despite the many strengths of this study, including the large and diverse sample, there are also some limitations. This was a cross-sectional study, which only allows for conclusions to be drawn about group differences at a single point in time. Longitudinal measurement invariance must be established before such a model can be used to draw conclusions about changes in cognitive functioning over time [13]. Our group is currently collecting longitudinal data from a community-based sample of Mexican Americans that will be used for such analyses, which should be a primary goal for future research in the area of cross-cultural neuropsychology. In addition, the current sample was recruited from a single geographic region, and the Hispanic participants were predominantly from a Mexican American background. One specific limitation of the five-factor model pertains to the breadth of the visuospatial and motor factors. The visuospatial factor is indicated by two test scores, CLOX1 and CLOX2, while the motor factor is indicated by four test scores, dominant and nondominant hand Grip Strength, with two trial scores per hand. As such, the model provides a narrowly focused ability estimate for these two cognitive domains, which may be undesirable in some assessment contexts.

Few neuropsychological tests have been designed *a priori* to provide unbiased estimates of cognitive functioning across diverse groups, and few existing test batteries have been subjected to *post hoc* validation for this purpose [13], [50]. Without confirmation that a test is essentially free from ethnic, racial, or linguistic bias, it is difficult to determine the relative contributions of true cognitive differences versus systematic error variance when interpreting observed test score differences. The present study therefore makes an important contribution to the literature by providing clinicians and researchers with another tool for generating valid cognitive outcome measures across two important dimensions of diversity.

**Research in Context**

1. Systematic review: We performed a literature search in PubMed and Scopus to identify articles that have investigated the factor structure of neuropsychological test batteries in older adults, and for studies that subjected these models to measurement invariance testing or similar methodological approaches (e.g., differential item functioning). Despite the growing ethnic and linguistic diversity in the United States, few neuropsychological tests used in dementia assessment are able to provide equally valid estimates of cognitive functioning across diverse groups.
2. Interpretation: The present study identified a set of 19 cognitive test variables that together provide an essentially unbiased estimate of five cognitive domains in Mexican Americans and non-Hispanic whites regardless of whether the tests were administered in Spanish or English.
3. Future directions: The current results support the use of the five-factor model reported here in future research seeking to investigate cognitive functioning in populations containing Mexican American and Spanish-speaking older adults.

## References

[1] Fletcher E., Gavett B., Harvey D., Farias S.T., Olichney J., Beckett L. (2018). Brain volume change and cognitive trajectories in aging. Neuropsychology.

[2] Salloway S., Sperling R., Fox N.C., Blennow K., Klunk W., Raskind M. (2014). Two phase 3 trials of Bapineuzumab in mild-to-moderate Alzheimer's disease. N Engl J Med.

[3] Salmon D.P., Bondi M.W. (2009). Neuropsychological assessment of dementia. Annu Rev Psychol.

[4] Morhardt D., Weintraub S., Khayum B., Robinson J., Medina J., O'Hara M. (2015). The CARE pathway model for dementia. Psychiatr Clin North Am.

[5] Hayden K.M., Reed B.R., Manly J.J., Tommet D., Pietrzak R.H., Chelune G.J. (2011). Cognitive decline in the elderly: An analysis of population heterogeneity. Age Ageing.

[6] Mungas D., Beckett L., Harvey D., Tomaszewski Farias S., Reed B., Carmichael O. (2010). Heterogeneity of cognitive trajectories in diverse older persons. Psychol Aging.

[7] Díaz-Venegas C., Downer B., Langa K.M., Wong R. (2016). Racial and ethnic differences in cognitive function among older adults in the USA: Cognition of US older adults by race/ethnicity. Int J Geriatr Psychiatry.

[8] Early D.R., Widaman K.F., Harvey D., Beckett L., Park L.Q., Farias S.T. (2013). Demographic predictors of cognitive change in ethnically diverse older persons. Psychol Aging.

[9] Manly J.J., Jacobs D.M., Sano M., Bell K., Merchant C.A., Small S.A. (1998). Cognitive test performance among nondemented elderly African Americans and whites. Neurology.

[10] Zahodne L.B., Stern Y., Manly J.J. (2015). Differing effects of education on cognitive decline in diverse elders with low versus high educational attainment. Neuropsychology.

[11] Schmitt N., Kuljanin G. (2008). Measurement invariance: Review of practice and implications. Hum Resour Manag Rev.

[12] Vandenberg R.J., Lance C.E. (2000). A review and synthesis of the measurement invariance literature: Suggestions, practices, and recommendations for organizational research. Organ Res Methods.

[13] Barnes L.L., Yumoto F., Capuano A., Wilson R.S., Bennett D.A., Tractenberg R.E. (2016). Examination of the factor structure of a global cognitive function battery across race and time. J Int Neuropsychol Soc.

[14] Millsap R.E., Olivera-Aguilar M. (2012). Investigating measurement invariance using confirmatory factor analysis. Handbook of Structural Equation Modeling.

[15] Meredith W. (1993). Measurement invariance, factor analysis and factorial invariance. Psychometrika.

[16] Park L.Q., Gross A.L., McLaren D.G., Pa J., Johnson J.K., Mitchell M. (2012). Confirmatory factor analysis of the ADNI neuropsychological battery. Brain Imaging Behav.

[17] Edwards M., Hall J., Williams B., Johnson L., O'Bryant S. (2016). Molecular markers of amnestic mild cognitive impairment among Mexican Americans. J Alzheimers Dis.

[18] Johnson L.A., Edwards M., Gamboa A., Hall J., Robinson M., O'Bryant S.E. (2017). Depression, inflammation, and memory loss among Mexican Americans: Analysis of the HABLE cohort. Int Psychogeriatr.

[19] Johnson L.A., Gamboa A., Vintimilla R., Cheatwood A.J., Grant A., Trivedi A. (2015). Comorbid depression and diabetes as a risk for mild cognitive impairment and Alzheimer's disease in elderly Mexican Americans. J Alzheimers Dis.

[20] Szerlip H.M., Edwards M.L., Williams B.J., Johnson L.A., Vintimilla R.M., O'Bryant S.E. (2015). Association between cognitive impairment and chronic kidney disease in Mexican Americans. J Am Geriatr Soc.

[21] Morris J.C., Heyman A., Mohs R.C., Hughes J.P., van Belle G., Fillenbaum G. (1989). The Consortium to Establish a Registry for Alzheimer's Disease (CERAD). Part I. Clinical and neuropsychological assessment of Alzheimer's disease. Neurology.

[22] Wechsler D. (1997). Wechsler Memory Scale - Third Edition (WMS-III).

[23] Wechsler D. (1997). Wechsler Adult Intelligence Scale - Third Edition (WAIS-III).

[24] Royall D.R., Mahurin R.K., Gray K.F. (1992). Bedside assessment of executive cognitive impairment: the executive interview. J Am Geriatr Soc.

[25] Reitan R., Wolfson D. (1993). The Halstead-Reitan Neuropsychological Test Battery: Theory and clinical applications.

[26] Kaplan E., Goodglass H., Weintraub S. (1983). The Boston Naming Test.

[27] Royall D.R., Cordes J.A., Polk M. (1998). CLOX: An executive clock drawing task. J Neurol Neurosurg Psychiatry.

[28] Lezak M.D., Howieson D.B., Bigler E.D., Tranel D. (2012). Neuropsychological Assessment.

[29] Strauss E., Sherman E.M.S., Spreen O., Spreen O. (2006). A compendium of neuropsychological tests: administration, norms, and commentary.

[30] Hu L., Bentler P.M. (1999). Cutoff criteria for fit indexes in covariance structure analysis: Conventional criteria versus new alternatives. Struct Equ Model Multidiscip J.

[31] Byrne B.M. (2012). Structural equation modeling with Mplus: basic concepts, applications, and programming.

[32] Satorra A., Bentler P.M. (2001 Dec). A scaled difference chi-square test statistic for moment structure analysis. Psychometrika.

[33] Muthén L., Muthén B. (1998). Mplus User's Guide.

[34] R Core Team (2017). R: A Language and Environment for Statistical Computing [Internet]. http://www.r-project.org/.

[35] Rosseel Y. (2012). lavaan: An R Package for Structural Equation Modeling. J Stat Softw.

[36] semTools Contributors (2016). semTools: Useful tools for Structural Equation Modeling [Internet]. https://CRAN.R-project.org/package=semTools.

[37] Raykov T., Marcoulides G.A. (2011). Introduction to Psychometric Theory.

[38] Nunnally J.C., Bernstein I.H. (1994). Psychometric Theory.

[39] O'Bryant S.E., Humphreys J.D., Schiffer R.B., Sutker P.B. (2007). Presentation of Mexican Americans to a memory disorder clinic. J Psychopathol Behav Assess.

[40] O'Bryant S.E., Leigh J., Valerie B., Melissa E., Robert B., Benjamin W. (2013). Characterization of Mexican Americans with mild cognitive impairment and Alzheimer's deisease. J Alzheimer Dis.

[41] O'Bryant S.E., Johnson L., Reisch J., Edwards M., Hall J., Barber R. (2013). Risk factors for mild cognitive impairment among Mexican Americans. Alzheimers Dement.

[42] Gavett B.E., Fletcher E., Harvey D., Farias S.T., Olichney J., Beckett L. (2018). Ethnoracial differences in brain structure change and cognitive change. Neuropsychology.

[43] O'Bryant S.E., Hall J.R., Cukrowicz K.C., Edwards M., Johnson L.A., Lefforge D. (2011). The differential impact of depressive symptom clusters on cognition in a rural multi-ethnic cohort: a Project FRONTIER study. Int J Geriatr Psychiatry.

[44] O'Bryant S.E., Xiao G., Barber R., Reisch J., Doody R., Fairchild T. (2010). A serum protein–based algorithm for the detection of Alzheimer disease. Arch Neurol.

[45] O'Bryant S.E., Guanghua X., Melissa E., Michael D., Bala G.V., Ralph M. (2013). Biomarkers of Alzheimer's disease among Mexican Americans. J Alzheimer Dis.

[46] O'Bryant S.E., Guanghua X., Fan Z., Melissa E., C G.D., Xiangling Y. (2014). Validation of a serum screen for Alzheimer's disease across assay platforms, species, and tissues. J Alzheimer Dis.

[47] Bowden S.C., Finch S., Bowden S.C. (2017). When is a test reliable enough and why does it matter?. Neuropsychological Assessment in the Age of Evidence-based Practice: Diagnostic and Treatment Evaluations.

[48] Hall J.R., Balldin V.H., Gamboa A., Edwards M.L., Johnson L.A., O'Bryant S.E. (2018). Texas Mexican American adult normative studies: Normative data for the Repeatable Battery for the Assessment of Neuropsychological Status (RBANS). Dev Neuropsychol.

[49] O'Bryant S.E., Edwards M., Johnson L., Hall J., Gamboa A., O'Jile J. (2018). Texas Mexican American adult normative studies: Normative data for commonly used clinical neuropsychological measures for English- and Spanish-speakers. Dev Neuropsychol.

[50] Mungas D., Widaman K.F., Reed B.R., Tomaszewski Farias S. (2011). Measurement invariance of neuropsychological tests in diverse older persons. Neuropsychology.

